# Comparison of ankle joint kinematics of a single athlete during an ankle inversion sprain incident and normal non-injury motions

**DOI:** 10.1186/1757-1146-7-S1-A28

**Published:** 2014-04-08

**Authors:** Zoe YS Chan, Sophia CW Ha, Daniel TP Fong, KM Chan

**Affiliations:** 1 Division of Biomedical Engineering, Department of Electronic Engineering, The Chinese University of Hong Kong, Hong Kong; 2 Department of Orthopaedics and Traumatology, Prince of Wales Hospital, Faculty of Medicine, The Chinese University of Hong Kong, Hong Kong; 3 School of Sport, Exercise and Health Sciences, Loughborough University, Leicestershire LE11 3TU, UK

## Introduction

The purpose of this study was to compare the ankle joint kinematics including the angles, and their respective angular velocities of a tennis player during an ankle sprain incident and normal non-injury motion. And to deduce whether the sideward cutting motion of the athlete is an intrinsic factor to an ankle sprain.

## Methods

Model-Based Image Matching (MBIM) motion analysis technique allows us to understand the leg movement quantitatively by analyzing the three-dimensional human motion. With validation, it has been used to obtain ankle kinematics during ankle sprain incidents in various sports [1]. In this study, a sideward cutting motion performed by a female athlete was compared against her injured incident reported in 2012 [2].

## Results

**Figure 1:**
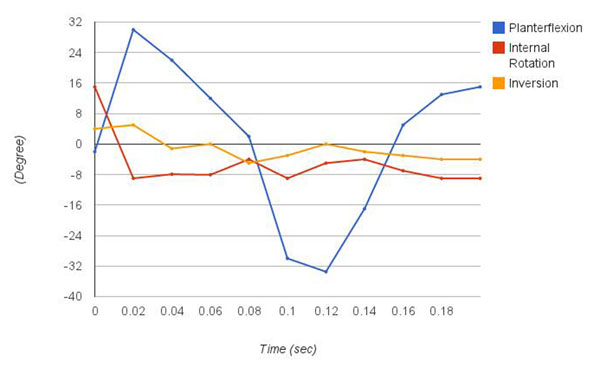
Profile of joint orientation. Joint orientation

**Figure 2:**
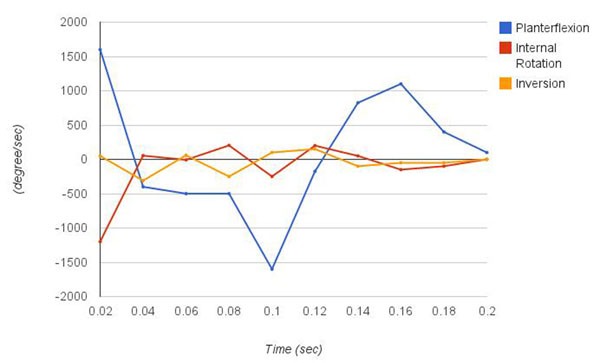
Profile of angular velocities. Joint velocity

Figure 1 and figure 2 show the right ankle kinematics profile of inversion, internal rotation, and plantarflexion during a sideward cutting motion to the right. Previously, the same athlete got injured performing a similar motion, regarding that incident, her peak inversion angle was reported to be 67^o^, which happened 0.17 second after foot strike [2]. The peak inversion angle of this case is 5°, significantly smaller compared to the injured case. The range of inversion angle was 5° eversion to 5° inversion. The degree of fluctuation of the angle of plantarflexion is greatest among the 3 planes of motion. It ranges from -33.5° to 30°. The peak velocity is 1600°/sec for both ways, doriflexion and plantarflexion.

## Conclusion

This study further demonstrates that the sideward cutting motion does not require internal rotation and inversion, instead, ankle goes from plantarflexed to doriflexed, and then back to plantarflexed in a short time. An inverted ankle orientation on landing could be the inciting event of an ankle sprain when performing similar motion. However, a rapid joint motion in the plantarflexion/doriflexion plane is not likely to cause an ankle sprain. Therefore, the risk of performing the sideward cutting depends mostly on the ankle orientation during landing.
